# A preliminary evaluation of targeted nanopore sequencing technology for the detection of *Mycobacterium tuberculosis* in bronchoalveolar lavage fluid specimens

**DOI:** 10.3389/fcimb.2023.1107990

**Published:** 2023-11-10

**Authors:** Xiaoke Sun, Jingchao Song, Xia Leng, Fuli Li, Haojie Wang, Jiaqian He, Wenhua Zhai, Zhenjing Wang, Qingqing Wu, Zheng Li, Xianglin Ruan

**Affiliations:** ^1^ Department of Tuberculosis, Henan Provincial Chest Hospital, Zhengzhou University, Zhengzhou, Henan, China; ^2^ Tuberculosis Clinical Research Center of Henan Province, Zhengzhou, Henan, China; ^3^ Thoracic Surgery Department, Department of Cerebral Surgery, Henan Provincial Chest Hospital, Zhengzhou University, Zhengzhou, China; ^4^ Department of Infectious Diseases, Xixian People's Hospital, Xixian, Xinyang, China; ^5^ Department of Endoscope Clinic, Henan Provincial Chest Hospital, Zhengzhou University, Zhengzhou, Henan, China; ^6^ Department of Laboratory Medicine, Henan Provincial Chest Hospital, Zhengzhou University, Zhengzhou, Henan, China

**Keywords:** targeted nanopore sequencing, *Mycobacterium tuberculosis*, bronchoalveolar lavage fluid, detection, pulmonary tuberculosis

## Abstract

**Objective:**

To evaluate the efficacy of targeted nanopore sequencing technology for the detection of *Mycobacterium tuberculosis*(*M.tb.*) in bronchoalveolar lavage fluid(BALF) specimens.

**Methods:**

A prospective study was used to select 58 patients with suspected pulmonary tuberculosis(PTB) at Henan Chest Hospital from January to October 2022 for bronchoscopy, and BALF specimens were subjected to acid-fast bacilli(AFB) smear, *Mycobacterium tuberculosis* MGIT960 liquid culture, Gene Xpert MTB/RIF (Xpert MTB/RIF) and targeted nanopore sequencing (TNS) for the detection of *M.tb.*, comparing the differences in the positive rates of the four methods for the detection of patients with different classifications.

**Results:**

Among 58 patients with suspected pulmonary tuberculosis, there were 48 patients with a final diagnosis of pulmonary tuberculosis. Using the clinical composite diagnosis as the reference gold standard, the sensitivity of AFB smear were 27.1% (95% CI: 15.3-41.8); for *M.tb* culture were 39.6% (95% CI: 25.8-54.7); for Xpert MTB/RIF were 56.2% (95% CI: 41.2-70.5); for TNS were 89.6% (95% CI: 77.3-96.5). Using BALF specimens Xpert MTB/RIF and/or *M.tb.* culture as the reference standard, TNS showed 100% (30/30) sensitivity. The sensitivity of NGS for pulmonary tuberculosis diagnosis was significantly higher than Xpert MTB/RIF, *M.tb.* culture, and AFB smear. Besides, P values of <0.05 were considered statistically significant.

**Conclusion:**

Using a clinical composite reference standard as a reference gold standard, TNS has the highest sensitivity and consistency with clinical diagnosis, and can rapidly and efficiently detect PTB in BALF specimens, which can aid to improve the early diagnosis of suspected tuberculosis patients.

## Introduction

Tuberculosis (TB) is an infectious disease caused by *M.tb.* and poses a major threat to human health. According to the World Health Organization (WHO) Global TB annual report in 2021, China estimates that there are about 780,000 TB cases and the estimated TB incidence rate is about 55 cases per 100,000 people. China ranks 3rd in estimated TB incidence among 30 high TB burden countries, below Indonesia and India. Pathogenic positive rate of TB in China is only 58% (World Health O, 2022). Therefore, rapid and accurate clinical diagnostic methods are essential to improve the diagnosis of tuberculosis, treat patients with tuberculosis, and prevent the spread of tuberculosis.

Nowadays, in the general testing clinical diagnosis method of *M.tb.*, that AFB smear has quick and easy test method, low cost advantage, but pathogen positivity rate is low, *M.tb.* incubation takes a long time, which cannot applicable to clinical rapid diagnosis. *M.tb.* culture-based phenotypic drug susceptibility testing (DST) is impacted by biosafety, long incubation time and the decreased incubation rate after application of anti-tuberculosis drugs. The slow-growing feature of Mycobacterium tuberculosis makes this methodology take a long time and uncertainty in results due to possible poor cultivation or microbial contamination which cannot fulfill the requirements of rapid clinical diagnosis. The rapid and accurately testing specimens for *M.tb.* and the rapid diagnosis of drug-resistant tuberculosis are essential for the effective control of drug-resistant tuberculosis epidemic. The drug-resistant phenotype in *M.tb.* is confirmed primarily by chromosomal mutations in several genes (Gygli et al., 2017). For example, rifampicin resistance caused by mutations in the *rpoB* gene encoding the β subunit of RNA polymerase is the most common gene mutation in *M.tb.* Xpert MTB/RIF assays for *M.tb.* and *rpoB* gene mutations directly from sputum using semi-nested PCR. Xpert MTB/RIF was initially recommended by WHO for TB diagnosis in 2010, however, Xpert MTB/RIF showed less sensitive test results for samples with low bacterial load. Moreover, the assay only detects rifampicin resistance mutations and cannot report the type of mutation.

With the rapidly development of Next-generation sequencing(NGS) in recent years, WHO recommends NGS for detection of drug resistance related mutations in the Mycobacterium tuberculosis complex(MTBC) and targeted high-throughput sequencing technology enables rapid detection of both common and rare genetic variations. Targeted NGS is focused on sequencing a select set of genes or gene regions that have known or suspected associations with a specific pathogen (e.g., *M.tb.*) or a specific phenotype (e.g. drug resistance)(World Health O, 2018). The method has the advantages of low sequencing cost, easy customization, high-throughput, rapid testing and rapid reporting of test results, and does not dependent on the *M.tb.* culture.

In this study, the performance of TNS in BALF specimens was prospectively evaluated, and the clinical diagnostic value for *M.tb.* was evaluated. This study also compared the differences between TNS and AFB smear, Xpert MTB/RIF, and *M.tb.* culture in the clinical diagnostic effectiveness of tuberculosis.

## Materials and methods

### Ethical approval

This study was approved by the Ethics Committee of Henan Provincial Chest Hospital, No. (2021) Ethics No. (11-04). Written informed consent was signed by all participants in this study.

### Patient recruitment

Patients with clinical diagnosis of pulmonary tuberculosis were enrolled prospectively and consecutively at Henan Chest Hospital from January to October 2022. Clinical diagnostic criteria for tuberculosis refer to the *WS196-2017 Classification of Tuberculosis* (China health industry, 2017) and *WS288-2017 Diagnostic Criteria for Tuberculosis* (Xia et al., 2018). Patients had undergone all routine clinical examinations before enrollment, including: chest CT, sputum smear for molecular assay, tuberculin skin test (TST), and *M.tb.* enzyme-linked immunospot (ELISPOT) assay. 10 patients with non-pulmonary tuberculosis included 4 cases of pulmonary Non-tuberculous mycobacteriosis, 3 cases of pneumomycosis, 1 case of pneumonia, 1 case of pulmonary sequestration and 1 case of nocardiosis. The diagnosis of the above patient was confirmed by bacteriological culture of the BALF, genetic testing of the BALF and postoperative pathological results.

### Inclusion and exclusion criteria

#### Inclusion criteria

Being aged 15–80 years; clinical symptoms related to pulmonary tuberculosis (e.g., cough, fever, hemoptysis); abnormal imaging of pulmonary tuberculosis; positive ELISPOT test or positive TST; patients have informed the purpose of the study and signed an informed consent form.

#### Exclusion criteria

Patient refusal to perform an invasive examination; bleeding tendency or coagulation disorders; severe cardiorespiratory dysfunction; The size of the specimens sent for testing cannot fulfill the testing requirements.

### Specimen collection

Patients were included in the bronchoscopy, and bronchoalveolar lavage was performed on the corresponding lesions in combination with the patient’s chest CT imaging performance: Saline was instilled into the bronchopulmonary segment of the lesion, and the BALF was recovered by negative pressure suction and placed in a sterile bottle, which could be repeated until 20mL of BALF was collected; the collected BALF was placed equally in tubes A, B, C, and D and sealed, and tube A was sent for antacid staining smear, tube B for tuberculosis culture, tube C for Xpert MTB/RIF assay, and tube D for TNS.

### Main equipment and test methods

#### AFB smear

N-acetyl-l-cysteine–NaOH was used for digestion and decontamination of the specimens, and auramine O, a fluorescentdye, was used for direct smear microscopy and confirmed by staining with Ziehl–Neelsen, based on the WHO guidelines.

#### *Mycobacterium tuberculosis* culture and phenotypic DST

N-acetyl-l-cysteine–NaOH for digestion and decontamination of specimens, Liquid medium (Bactec MGIT 960 Mycobacterial Culture System) was used for Mycobacterium tuberculosis culture. All MTB isolates were validated by both a growth test on p-nitrobenzoic acid containing medium and an MBP 64 antigen detection kit. Non-tuberculosis mycobacteria (NTM) were excluded. Antimicrobial susceptibility testing (AST) use MicroDST™ test kit. All steps were performed by trained and specialized staff in a biosafety cabinet in accordance with the relevant guidelines. The critical concentrations were 0.2µg/mL for isoniazid, 2.0µg/mL for streptomycin, 5.0µg/mL for ethambutol, 1.0µg/mL for rifampin, 2.0µg/mL for ofloxacin, 0.5µg/mL for moxifloxacin,2.0μg/mL for capreomycin, 1.0µg/mL for amikacin, 2.5μg/mL for prothionamide, 2.0μg/mL for aminosalicylic acid, respectively. Pyrazinamide phenotypic DST was performed using the Middlebrook 7H10 agar medium. The critical concentration was 100.0µg/mL for pyrazinamide. The liquid culture-positive strains were extracted, adjusted to the specified concentrations, then inoculated into drug-containing medium and control medium to be cultured at 36°C ± 1°C for 14 days, drug susceptibility was determined by the number of colonies grown on the drug-containing medium and the number of colonies grown on the control medium(Baso, Zhuhai, China).

#### Xpert MTB/RIF

Mix the centrifuged lavage solution specimen with 2ml of sample processing solution. After stirring for 20 seconds, the mixed solution was incubated at room temperature for 15 minutes. Then, the first generation Xpert MTB/RIF reaction cassette is added to the 2ml of the mixed solution; the cassette is then placed into the test module for automated testing. The system automatically reads the MTB test results within 2 hours(Cepheid, Sunnyvale, CA, USA).

#### Targeted nanopore sequencing

200μL sample was taken into an EP tube and an equal volume of DTT solution, 10μL of proteinase K, 5μL of lysozyme, and 0.05mm Zirconium oxide grinding beads were added, and ground using a grinder. The supernatant was used for nucleic acid extraction by using QIAamp DNA Microbiome Kit (Qiagen). The concentration of extracted DNA was determined by dsDNA Assay Kit (ThermoFisher). The extracted DNA was amplified by multiplex PCR. The PCR product was purified by magnetic beads, and the eluted product was taken for qubit quantitative quality control. Equal quality PCR products with different barcode labels were pooled, and the pooled library was followed by the nanopore ligation kit. Nanopore sequencing and data analysis: 1) 100ng of the final prepared library was taken for up-sequencing; 2) the GridION platform was used for sequencing and MinKNOW software was used to collect real-time sequencing data; 3) the original sequencing reads were first-quality filtered and then subsequently analyzed, and sequenced fragments smaller than 200bp were removed. Host DNA reads were also removed by aligning to the human reference genome (GRCh38). 4) The remaining filtered reads were subsequently compared with the TB drug resistance gene database to generate drug resistance gene analysis results. TNS detects multiple anti-tuberculosis drugs resistance genes simultaneously. The drug resistance target genes of *M.tb.* tested by TNS are shown in **Table 1**.

**Table 1:** List of target genes sequenced by targeted nanopore, detected target gene mutation sites and mutation frequencies.

Drug	Target gene	Mutations	No. of isolates
**Rifampicin**	***rpoB***	**Leu511Pro**	1
		**Ser531Leu**	3
		**His526Tyr, Ser531Leu**	1
		**Thr400Ala, Ser531Leu**	1
		**Gln513Pro**	1
		**His526Tyr**	1
**Isoniazid**	***katG***	**Ser315Thr**	5
		**Thr394Ala, Ser315Thr**	1
	***inhA***	**– 15 C→T**	1
	***ahpC***		0
**Pyrazinamide**	***pncA***	**Asp63Gly**	1
		**Thr76Ile**	1
		**His51Arg**	1
**Ethambutol**	***embB***	**Tyr319Ser**	1
		**Asp328Tyr**	1
**Fluoroquinolones**	***gyrB***	**Asp94Gly**	1
		**Ala90Val**	1
		**Ser91Pro**	1
**Streptomycin**	***rpsl***	**Lys43Arg**	4
	***rrs***		0
**Prothionamide**	***inhA***		0
**Amikacin**	***rrs***		0
**Capreomycin**	***rrs***		0
**Aminosalicylic acid**	***folC***		0
	***thyA***		0
	***ribD***		0

### Statistical analysis

In this study, statistical analysis was performed using R (version 4.1.3). Using the clinical composite reference standard as the reference standard, the compliance rate was calculated = (number of true positive cases + number of true negative cases)/total number of tests × 100%; sensitivity = number of true positive cases/(number of true positive cases + number of false negative cases) × 100%; specificity = number of true negative cases/(number of true negative cases + number of false positive cases) × 100%. We evaluated the concordance between diagnostic assays with McNemar’s test. A p-value <0.05 was considered statistically significant for all the analyses.

## Results

### Clinical characteristics

**Figure 1:**
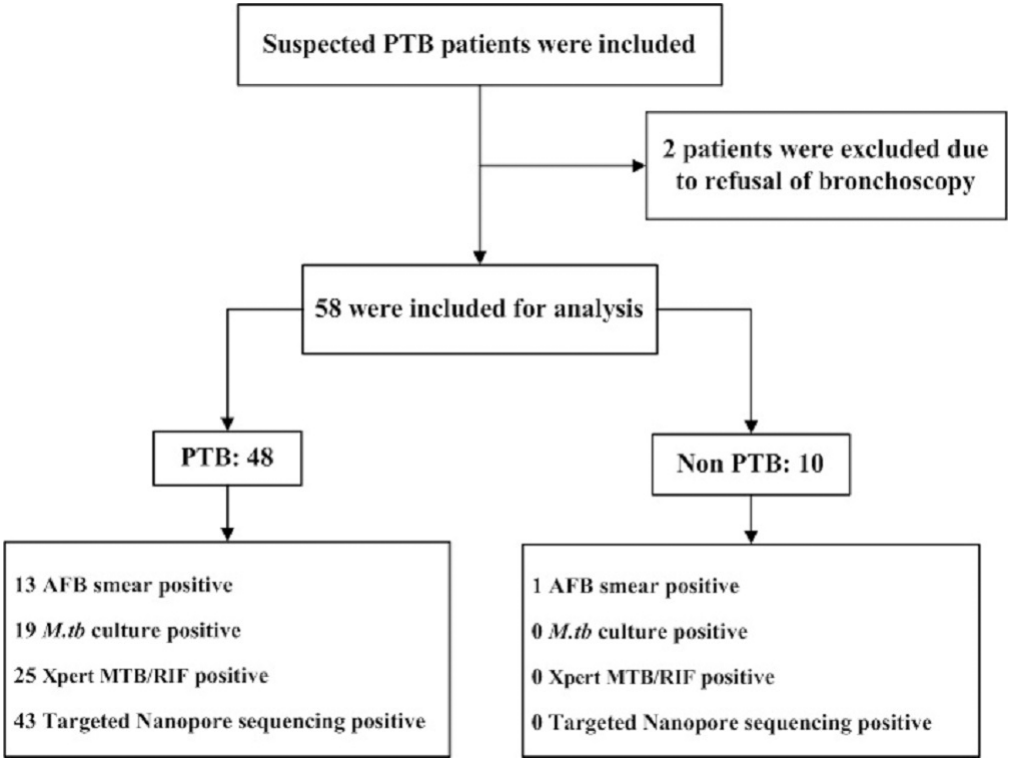
The classification flowchart of patients included in this study.

A total of 60 patients were enrolled between January and October 2022, excluding 2 patients who refused to perform bronchoscopy. The final enrollment of 58 patients was performed by TNS. **Figure 1** shows the classification of patients included in this study. Patients provide one BALF specimen each, there is no HIV in the patient. Clinical characteristics and demographics are shown in **Table 2**.

**Table 2:** Clinical characteristics of the included patients.

Clinical characteristics	All (n = 58)	PTB (n = 48)	Non-PTB (n = 10)
Age(year, mean ± SD)	44.60 ± 19.03	42.48 ± 19.77	54.80 ± 10.54
Gender			
Male(n, %)	40 (68.97)	33 (68.75)	7(70.00)
Female(n, %)	18( 31.03)	15 (31.25)	3(30.00)
TST			
Positive	51 (87.93)	46 (95.83)	5(50.00)
ELISPOT			
Positive	49 (84.48)	44 (91.67)	5(50.00)
HIV			
Positive	0 (0.00)	0 (0.00)	0(0.00)
Complaint symptoms			
asymptomatic	9 (15.52)	8 (16.67)	1(10.00)
hemoptysis	8 (13.79)	7 (14.58)	1(10.00)
cough	22 (37.93)	19 (39.58)	3(30.00)
fever	16 (27.58)	12 (25.00)	4(40.00)
chest tightness	3 (6.17)	2 (4.17)	1(10.00)

TST, tuberculin skin test; ELISPOT, enzyme-linked immunospot; HIV, Human immunodeﬁciency virus.

### Consistency between targeted nanopore sequencing test and other confirmatory diagnosis methods

**Figure 2:**
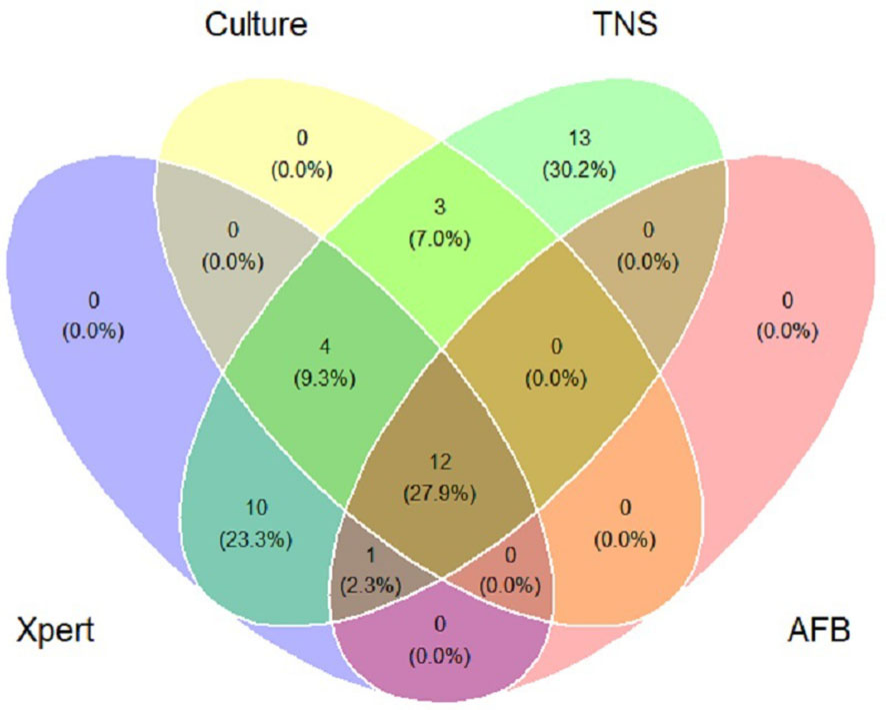
Venn diagram of positive tests for patients of pulmonary tuberculosis. Xpert: Xpert MTB/RIF; Culture: *Mycobacterium tuberculosis* culture; TNS: Targeted nanopore sequencing; AFB: acid-fast bacilli smear.

In this study, 30 patients with a final diagnosis of definite PTB (definite PTB was confirmed by either a positive Xpert MTB/RIF and/or *M.tb.* culture). TNS had a sensitivity of 100% (30/30) for definite PTB. *M.tb.* culture and Xpert MTB/RIF negative PTB cases were reported positive by TNS in 13 cases and were consistent with clinical diagnosis, **Table 3** showed the consistency between TNS test and other confirmatory diagnosis methods among patients with PTB. **Figure 2** shows the distribution and overlap of AFB smear, *M.tb.* culture, Xpert MTB/RIF, and TNS.

**Table 3:** Consistency between targeted nanopore sequencing test and other confirmatory diagnosis methods.

confirmatory diagnosis methods		targeted nanopore sequencing		P value
		Positive (N=43)	Negative (N=5)	
**GeneXpert MTB/RIF**	**Positive**	**27 (100.0%)**	**0 (0.0%)**	**<.001**
	**Negative**	**16 (76.2%)**	**5 (23.8%)**	
***Mycobacterium tuberculosis* Culture**	**Positive**	**19 (100.0%)**	**0 (0.0%)**	**<.001**
	**Negative**	**24 (82.8%)**	**5 (17.2%)**	
**GeneXpert MTB/RIF and/or *Mycobacterium tuberculosis* Culture**	**Positive**	**30 (100.0%)**	**0 (0.0%)**	**<.001**
	**Negative**	**13 (72.2%)**	**5 (27.8%)**	

### Diagnostic accuracy of the four tests

Using the clinical composite diagnosis as the reference gold standard, the sensitivity of AFB smear were 27.1% (95% CI: 15.3-41.8); for *M.tb* culture were 39.6% (95% CI: 25.8-54.7); for Xpert MTB/RIF were 56.2% (95% CI: 41.2-70.5); for TNS were 89.6% (95% CI: 77.3-96.5). **Table 4** shows the sensitivity, specificity, PPV, and NPV data for AFB smear, *M.tb.* culture, Xpert MTB/RIF, and TNS of BALF specimens, respectively. In these four testing methods, AFB smears showed the lowest diagnostic accuracy. TNS had the highest diagnostic accuracy and was obviously better than *M.tb.* culture and Xpert MTB/RIF (P<0.05). Xpert MTB/RIF and *M.tb.* culture each had moderate diagnostic accuracy, there is no statistical difference between them (P>0.05).

**Table 4:** The diagnostic accuracy of the four tests for the diagnosis of pulmonary tuberculosis.

Test	Sensitivity(%)	Specificity(%)	PPV(%)	NPV(%)
**AFB smear**	27.1 (95% CI: 15.3-41.8)	90.9 (95% CI: 58.7-99.8)	92.9 (95% CI: 66.1-99.8)	22.2 (95% CI: 11.2-37.1)
***M.tb* culture**	39.6 (95% CI: 25.8-54.7)	100.0 (95% CI: 69.2-100.0)	100.0 (95% CI: 82.4-100.0)	25.6 (95% CI: 13.0-42.1)
**Xpert MTB/RIF**	56.2 (95% CI: 41.2-70.5)	100.0 (95% CI: 69.2-100.0)	100.0 (95% CI: 87.2-100.0)	32.3 (95% CI: 16.7-51.4)
**Targeted nanopore sequencing**	89.6 (95% CI: 77.3-96.5)	100.0 (95% CI: 69.2-100.0)	100.0 (95% CI: 91.8-100.0)	66.7 (95% CI: 38.4-88.2)

AFB, acid-fast bacilli; CI, confidence interval; M.tb, Mycobacterium tuberculosis; NPV, negative predictive value; PPV, positive predictive value.

### Mutation sites and frequencies of relevant drug resistance genes detected by targeted nanopore sequencing

Among the patients who tested positive for TNS, 10 patients had different targeting gene mutations in *M.tb.*, Five of them tested positive for *M.tb.* culture and five tested negative for *M.tb.* culture. The relevant drug resistance gene mutation sites and frequencies detected by TNS are shown in **Table 1**. The *rpoB* gene detected amino acid codon mutations at 511, 513, etc. The *katG* gene detected the most codon mutations at position 315. The *pncA* gene detected codon mutations at positions 63 and 76. The *embB* detected codon mutations at positions 319 and 328. The *gyrB* gene detected codon mutations at positions 90, 91, and 94, which is highly consistent with fluoroquinolone resistance. The *rpsL* gene detected codon mutations at position 43.

### Targeted nanopore sequencing compared with phenotypic DST

All 19 patients with TB positive cultures were subsequently tested for phenotypic drug susceptibility. We compared the genotypic and phenotypic drug susceptibility results of the 11 antitubercular drugs.The consistency analysis of the two DST results is shown in **Table 5**. Phenotypic and genotypic DST of rifampicin, isoniazid, streptomycin, and fluoroquinolones had high consistency.1 case of phenotypic resistance to ethambutol, no mutation detected in TNS. 1 case of phenotypic drug susceptibility to pyrazinamide and ethambutol, *pncA* and *embB* mutation in coding gene detected by TNS.

**Table 5:** Comparing genotypic drug susceptibility results of targeted nanopore versus phenotypic drug susceptibility.

Drug	Target genes & mutation sites		Phenotypically susceptible (No. of isolates)	Phenotypically resistant (No. of isolates)	Concordance (%)
Rifampicin	*rpoB*	Leu511Pro	0	1	100
	*rpoB*	Ser531Leu	0	2	
	*rpoB*	Ser531Leu, Thr400Ala	0	1	
	No mutations detected		15	0	
Isoniazid	*katG*	Ser315Thr	0	3	100
	*inhA*	– 15 C→T	0	1	
	No mutations detected		15	0	
Fluoroquinolones	*gyrB*	Asp94Gly	0	1	100
	*gyrB*	Ala90Val	0	1	
	No mutations detected		17	0	
Streptomycin	*rpsl*	Lys43Arg	0	3	100
	No mutations detected		16	0	
Pyrazinamide	*pncA*	Asp63Gly	1	0	94.7
	*pncA*	Thr76Ile	0	1	
	No mutations detected		17	0	
Ethambutol	*embB*	Tyr319Ser	1	0	89.5
	No mutations detected		17	1	
Amikacin	No mutations detected		19	0	-
Capreomycin	No mutations detected		19	0	-
Aminosalicylic acid	No mutations detected		19	0	-
Prothionamide	No mutations detected		19	0	-

## Discussion

In this study, we evaluated the application of TNS in the diagnosis of pulmonary TB. BALF specimens were selected and compared with Xpert MTB/RIF, AFB smear, and *M.tb.* culture, and it was found that the positive detection rate of TNS was significantly higher than that of the other three methods, and the differences were statistically significant. TNS also has high sensitivity in patients with negative results from Xpert MTB/RIF tests and from TB culture tests. Therefore, for patients with negative sputum smear or low sputum volume, the detection rate of TB patients can be significantly improved by BALF examination used in conjunction with TNS. This is very significant for controlling the TB epidemic.

In this study, more than 10 target genes were selected for TNS, covering most of the target genes for antituberculosis drug action. In contrast to Xpert MTB/RIF, which can only detect an 81bp region of the *rpob* gene, TNS can detect drug resistance genes of longer length, such as covering the full length of the *rpob* gene. Xpert MTB/RIF was unable to distinguish between heterogeneous drug resistance and silent mutations, but TNS can distinguish them and identify them better. The genotypic and phenotypic drug susceptibility results from TNS also showed high concordance, but the genotypic drug susceptibility from TNS showed 1 month earlier than the phenotypic drug susceptibility. It is very valuable for guiding clinicians’ treatment.

In this study, the mutation sites detected in the target genes of anti-TB drugs in routine use were consistent with the reported previous studies. *rpoB* gene detected amino acid codon mutations at 531 was the most common, and was highly associated with rifampicin resistance, the test results are consistent with the findings of Williams DL (Williams et al., 1998; Nakata et al., 2012). The *katG* gene detected the most codon mutations at position 315, the test results are consistent with the reported by Wengnack NL (Wengenack et al., 1997). The *rpsL* gene detected codon mutations at position 43, which is consistent with that reported by He J (He et al., 2014).

In this study, we compared the results of 19 cases of genotypic and phenotypic drug susceptibility only, which may have biased the concordance between genotypic and phenotypic drug susceptibility. This is because the number of cases with positive TB cultures and the number of drug-resistant TB cases included was low. In a follow-up study, we plan to increase the sample size and the number of TB-resistant patients for more thorough subgroup and in-depth analysis. We combined the genotypic and phenotypic drug susceptibility results in the subsequent clinical treatment, and the treatment was effective after adjusting the anti-tuberculosis regimen. In particular, phenotypic DST for pyrazinamide is not reliable (Piubello et al., 2018), so genotypic DST can compensate for the lack of phenotypic DST.

WHO published *The use of next-generation sequencing technologies for the detection of mutations associated with drug resistance in Mycobacterium tuberculosis complex: technical guide* in 2018. This guideline demonstrates that next-generation sequencing is a powerful tool to ability to improve TB management and control through rapid and accurate detection of all clinically relevant genetic mutations, leading to rapid diagnosis of drug-resistant TB bacteria in clinical specimens. This information is critical for clinicians to rapidly decide on the best therapy to treat multiple and extensively drug-resistant TB. NGS can be successfully used as a highly efficient diagnostic technique for drug-resistant TB and its performance is expected to improve as we increasingly understand the genetic basis of phenotypic resistance and the relevance of genetic resistance to the clinic.

Next-generation sequencing (NGS) has great potential as a rapid diagnostic method for drug-resistant TB in different clinical laboratory settings worldwide. While current molecular assays for drug-resistant TB primarily identify limited *M.tb.* drug-resistant mutations indirectly by hybridizing probes to specific gene sequences, NGS assays can provide detailed sequence information for multiple gene regions or entire genomes. Advances in NGS technology have made possible the routine application of targeted NGS and whole-genome sequencing (WGS) in the *Mycobacterium tuberculosis* complex, and WGS can provide a nearly complete of the *Mycobacterium tuberculosis* genome, but WGS is usually performed only on culture-positive strains because relatively high-quality DNA is required to generate complete WGS data. Therefore, WGS is more suitable for epidemiological investigations and various scientific studies. Targeted NGS, on the other hand, can generate *Mycobacterium tuberculosis* sequence data at specific genetic loci of interest. Since *M.tb* resistance arises mainly through point mutations in specific genetic targets, targeted NGS offers great promise for the rapid diagnosis of drug-resistant TB. Targeted NGS is achieved by analysis of drug-resistant gene amplification or hybridization/capture, allowing large sequence coverage depth for sequencing large targets. The large depth of coverage provides high confidence in mutation detection and also enables the detection of heterogeneous drug resistance in samples. Targeted NGS may yield valid results even for minimal amounts and quality of DNA input, and the technique has been successfully applied to drug resistance detection in clinical TB specimens, further reducing the time to diagnosis of TB and drug-resistant TB (World Health O, 2018).

The nanopore sequencing platform, developed by Oxford Nanopore Sequencing Technologies (ONT) (Oxford, UK), identifies DNA bases by measuring the change in conductivity generated as the DNA strand passes through a biological nanopore (MinION, 2022), which has a slightly shorter sample and library preparation steps and generally lower infrastructure requirements compared to Illumina technology. Nanopore sequencing is also increasingly used in clinical applications due to its high throughput, real-time readability, and speed of data analysis. Nanopore sequencing is increasingly being used in TB, with previous studies demonstrating its excellent accuracy in predicting *M.tb* resistance. Mariner-Llicer C et al. compared the ability of the Illumina platform and MinION sequencing platform for rapid detection of *Mycobacterium tuberculosis* drug resistance genes and found that the nanopore sequencer-based targeted sequencing approach and the Illumina platform whole genome sequencing approach. The results of the nanopore sequencing-based method and the Illumina platform whole-genome sequencing method was 100% consistent in detecting drug-resistant mutations in *Mycobacterium tuberculosis*(Mariner-Llicer et al., 2021). Tafess K et al. used a second-generation sequencing Miseq and a third-generation nanopore sequencing MinIon to target 19 genes associated with drug resistance in *Mycobacterium tuberculosis*. The average sensitivity of second-generation sequencing was 82.3% and specificity was 98.0%; nanopore sequencing had 94.8% sensitivity and 98.0% specificity, showing that nanopore sequencing covered more regions of *Mycobacterium tuberculosis* drug resistance genes with faster detection and higher detection rate (Tafess et al., 2020). The study by Guocan Yu et al. evaluated the effectiveness of nanopore sequencing of respiratory specimens in early diagnosis of pulmonary tuberculosis (PTB) while comparing it with *M.tb* culture and Xpert MTB/RIF. The sensitivity and specificity of AFB smear were 27.6% and 87.5%, respectively; the sensitivity and specificity of *M.tb.* culture were 57.8% and 100.0%, respectively; the sensitivity and specificity of Xpert MTB/RIF were 62.9% and 97.9%, respectively; and the sensitivity and specificity of nanopore sequencing were 94.8% and 97.9%, respectively, which proved that the diagnostic accuracy of nanopore sequencing for PTB The sensitivity and specificity of nanopore sequencing were 94.8% and 97.9%, respectively. Nanopore sequencing can replace Xpert MTB/RIF for the initial detection of PTB and improve the accuracy of TB diagnosis (Yu et al., 2022).

This study is exploratory and has some limitations. First, because of the limited study funding, the number of samples collected was small, which may have a biasing effect on the results, but the advantages of TNS in the etiological detection of pulmonary tuberculosis were observed from the cases included in the study. Secondly, although NGS technology can also predict drug resistance of Mycobacterium tuberculosis, the number of drug-resistant patients in the study selection is not sufficient for comparative analysis, and future studies should increase the sample size and the number of drug-resistant TB samples for more detailed grouping and in-depth analysis.

China revised the diagnostic criteria for tuberculosis in 2017, using positive results from molecular biology techniques as diagnostic criteria for pathogenically positive tuberculosis, greatly complementing traditional bacteriological detection methods (National Health, 2017). This study evaluates the potential value of TNS for the diagnosis of tuberculosis, and the application of BALF specimens for TNS has good accuracy for the diagnosis of tuberculosis, significantly better than Xpert MTB/RIF, AFB smear, and *M.tb.* culture. And the results were highly consistent with the phenotypic DST and Xpert MTB/RIF, and the results of multi-drug resistance detection could be obtained within 48 hours. With the further development and improvement of NGS technology, it gradually achieves low cost and high efficiency and is expected to become a new viable method for clinical research and diagnosis of TB and drug-resistant TB.

## Data availability statement

The original contributions presented in the study are included in the article/supplementary materials, further inquiries can be directed to the corresponding author/s.

## Author contributions

XS, XL, FL, JS and XR designed and supervised the study. XS, QW, ZW enrolled patients and assisted in the collection of samples. XS, HW, JH, WZ performed experiments and analyzed data. XS, XR wrote and edited the manuscript. All authors contributed to the article and approved the submitted version.
